# Timing of Surgery for Hip Fracture in Patients on Direct Oral Anti-coagulants: A Population-Based Cohort Study

**DOI:** 10.1177/21514593221088405

**Published:** 2022-03-26

**Authors:** En Lin Goh, Swathikan Chidambaram, Suprabha Rai, Angela Kannan, Sambandam Anand

**Affiliations:** 1 Oxford Trauma, Nuffield Department of Orthopaedics, Rheumatology and Musculoskeletal Sciences, Kadoorie Centre, 6396 University of Oxford, Oxford, UK; 2 Oxford University Clinical Academic Graduate School, Medical Sciences Division, 6396 University of Oxford, Oxford, UK; 3 Department of Trauma, Horton General Hospital, 6397 Oxford University Hospitals NHS Foundation Trust, Oxford, UK

**Keywords:** fragility, fractures, pharmacology, osteoporosis, geriatric medicine, geriatric trauma

## Abstract

**Background:**

In patients with hip fracture on direct oral anti-coagulants (DOACs), timely surgery is important in optimising outcomes but the safety of early surgery is unclear. This study aims to evaluate the timing of surgery on peri- and post-operative outcomes in patients with hip fracture on DOAC therapy.

**Methods:**

Single-centre, retrospective, population-based cohort study of patients on DOAC therapy compared to standard care with low-molecular-weight heparin (LMWH) undergoing surgery for hip fracture. Data obtained: patient demographics, fracture classification, American Society of Anaesthesiologists (ASA) classification, time to surgery, procedure performed, type of DOAC, timing of last DOAC dose, use of reversal agents or pro-coagulants and length of stay. Outcomes assessed: pre- and post-operative haemoglobin levels, incidence of blood transfusion, major haemorrhage, venous thromboembolism (VTE) and death within 30 days of surgery.

**Results:**

A total of 755 patients were included. Compared to standard treatment, DOAC use was associated with a similar change in pre- and post-operative haemoglobin levels (*P* = .90), risk of blood transfusion (RR: 1.04, 95% CI: .70–1.54, P = .84), haemorrhage (RR: 1.51, 95% CI: .53-4.28, P = .44), VTE (RR: .92, 95% CI: .12–7.20, *P* = .94) and mortality (RR: 1.85, 95% CI: .89–3.84, *P* = .10), all of which were independent of the timing of surgery.

**Conclusion:**

This study builds on growing evidence that surgery for hip fracture in patients on DOAC therapy is not associated with an excessive risk of haemorrhage, irrespective of the timing of surgery. Timely surgical fixation of the hip fracture in this population is indicated in the absence of other risk factors for haemorrhage.

## Introduction

The use of direct oral anti-coagulants (DOACs) for anti-coagulation in stroke and venous thromboembolism (VTE) prophylaxis has grown extensively in recent years.^1^ Compared to existing anti-coagulants, DOACs are advantageous as they are administered orally, have a predictable pharmacological profile and do not require monitoring.^2^ Correspondingly, the widespread use of DOACs has led to an increasing number of patients with hip fracture receiving DOAC therapy.^1^ In these patients, DOAC use has the potential to delay surgery and increase the risk of haemorrhage, especially in the absence of a readily available reversal agent.^3^ As such, the British Committee for Standards for Haematology recommends that major surgery such as arthroplasty or internal fixation for hip fracture should take place at least 48 hours after the last DOAC dose.^4^

Patients with hip fracture are at risk of various complications, which increase morbidity and mortality, and timely surgery is important in optimising outcomes.^5,^^6^ Guidelines for hip fracture management in the United Kingdom advocate for surgery on the day of or the day after the injury.^7^ Surgery within 36 hours forms a key component of the requirement for the conditional component of the Department of Health’s Best Practice Tariff (BPT) for fragility hip fractures paid to National Health Service (NHS) providers.^8^ It is therefore important to balance the risks of peri-operative haemorrhage from early surgery and post-operative complications from delayed surgery in patients with hip fracture on DOAC therapy. However, there is a lack of evidence on the safety of the timing surgery in this population. This study aims to evaluate the timing of surgery on peri- and post-operative outcomes in patients with hip fracture on DOAC therapy.

## Methods

This study has been reported in accordance to the Strengthening the Reporting of Observational Studies in Epidemiology (STROBE) statement using the STROBE checklist (Supplemental material).^9^

### Population

Single-centre, retrospective, population-based cohort study of patients on DOAC therapy or standard care involving low-molecular-weight heparin (LMWH) undergoing surgery for hip fracture from 1 January 2016 to 31 December 2019. Patients with any of the following were excluded: missing data, age <65 years, warfarin use, previous major haemorrhage or inherited disorder of coagulation. All patients were followed up for 30 days post-operatively.

### Clinical Parameters

The following data was obtained: patient demographics, fracture classification, American Society of Anaesthesiologists (ASA) classification, time to surgery, procedure performed, type of DOAC, timing of last DOAC dose, use of reversal agents or pro-coagulants and length of stay. The outcomes assessed were pre- and post-operative haemoglobin levels, incidence of blood transfusion, major haemorrhage, pulmonary embolism (PE) and deep vein thrombosis (DVT), and death within 30 days of surgery. Major haemorrhage was defined in accordance to the International Association of Thrombosis and Haemostasis Scientific Committee and included haemorrhage from the gastrointestinal, urinary and cerebrovascular systems.^10^ In the event of death, the coroner’s report, hospital and general practice records were reviewed to establish the cause of death.

### Peri-Operative Anti-coagulation Regime

The administration regime for all medications was based on guidelines from the British National Formulary. All DOACs were stopped on admission and restarted as described subsequently. Apixaban was administered 2.5 mg twice daily to be started 12–24 hours after surgery. Rivaroxaban was administered 10 mg once daily to be started 6–10 hours after surgery. Dabigatran was administered at 75 mg, to be taken 1–4 hours after surgery, followed by 150 mg once daily for 10 days, to be taken on the first day after surgery. Dalteparin was administered initially at 5000 units for 1 dose, to be given on the evening before surgery, followed by 5000 units after 24 hours, then 5000 units every 24 hours.^11^

### Statistical Analysis

Statistical analysis was performed using IBM SPSS Statistics 24 (Armonk, New York). Summary statistics are presented as percentages, means and standard deviations. Multivariate and univariate analyses were performed using analysis of variance (ANOVA), Student t test, and Chi-squared test. Sensitivity analysis using multivariate logistic regression modelling was performed to identify effect size of inter-group variables. Relative risk (RR) is presented with 95% confidence interval (CI) and *P*-value and calculated using the method described by Altman.^12^

### Sample Size Estimate

The minimum clinically important difference (MCID) for the primary outcome measures reported in this study were calculated using the method described by Cohen.^13^ Based on these calculations, an estimated ratio of patients in the DOAC group to patients in the standard care group of 1:6.5 would be required to detect any MCIDs in change in pre- and post-operative haemoglobin levels, incidence of blood transfusion and major haemorrhage. This meant that the standard care group would require at least 526 patients. To minimise potential selection bias, the entire cohort of patients receiving standard care across the study duration was included.

### Ethics Approval

This was a retrospective study using only anonymised data previously acquired as part of the patient clinical work-up or for service evaluation purposes. Thus, ethical approval was waived following review of the study proposal by the local ethics committee.

## Results

A total of 755 patients were included in this study, with 81 patients on DOAC therapy and 674 patients on standard care (Table 1). The mean age of the study cohort was 83.4 ± 8.5 years. The number of males and females were 214 (28.3%) and 541 (71.7%), respectively. Although females made up the majority of patients in both groups, there was a significantly larger proportion of males in the DOAC group compared to the standard treatment group. Compared to the standard treatment group, patients in the DOAC group had significantly worse co-morbidities. The right hip was involved in 359 (47.5%) cases and the left hip in the remaining 396 (52.5%). Intracapsular fractures were the most common fracture type, occurring in 465 (61.6%) cases. The most common procedure was hemiarthroplasty, which was performed in 301 (39.9%) cases. The mean time to surgery from admission was 20.5 ± 13.7 hours and the mean length of stay was 15.9 ± 14.1 days. The majority of patients in both groups underwent surgery within 24 hours of admission. Apixaban was the most frequently prescribed DOAC in 56 (69.1%) patients followed by rivaroxaban in 18 (22.2%) patients. In the DOAC group, the mean time to surgery from last DOAC dose was 31.3 ± 12.2 hours, with 19 (23.5%) patients undergoing surgery within 24 hours of the last DOAC dose. Tranexamic acid was administered in 8 (9.9%) patients in the DOAC group and was more likely to be used in patients undergoing surgery within 24 hours of the last DOAC dose.

**Table 1.** Baseline demographics and clinical characteristics of the study population.

Characteristic	DOAC Group (n = 81)	Standard Treatment Group (n = 674)	*P-*value
Age – years	84.8 ± 7.7	83.2 ± 8.6	.10
Gender (%) Male Female	34 (42.0) 47 (58.0)	180 (26.7) 494 (73.3)	.01
ASA classification (%) Grade <3 Grade ≥3	8 (9.9) 73 (90.1)	191 (28.3) 483 (71.7)	.01
Laterality (%) Right Left	35 (43.2) 46 (56.8)	324 (48.1) 350 (51.9)	.41
Fracture classification (%) Intracapsular Intertrochanteric – grade A1/A2 Intertrochanteric – grade A3 Subtrochanteric	49 (60.5) 29 (35.8) 3 (3.7) 0 (.0)	416 (61.7) 209 (31.0) 30 (4.5) 18 (2.7)	.75
Procedure (%) Total hip arthroplasty Hemiarthroplasty Dynamic hip screw Intramedullary nail Cannulated screw fixation	2 (2.5) 38 (46.9) 33 (40.7) 3 (3.7) 5 (6.2)	75 (11.2) 263 (39.0) 281 (41.7) 29 (4.3) 26 (3.9)	.11
Time from admission to surgery – hours <24 ≥24	22.1 ± 11.8 55 (67.9) 26 (32.1)	20.3 ± 14.0 491 (72.8) 183 (27.2)	.35
Time from last dose to surgery – hours <24 ≥24	31.3 ± 12.2 19 (23.5) 62 (76.5)	–	–
DOAC typeApixaban Rivaroxaban Dabigatran Edoxaban	56 (69.1) 18 (22.2) 6 (7.4) 1 (1.2)	–	–
Tranexamic acid (%) <24 hours from last dose to surgery ≥24 hours from last dose to surgery	8 (9.9) 3 (15.8) 5 (8.1)	–	–
Length of stay – days	19.1 ± 14.2	15.6 ± 14.1	.04

**Figure 1.**
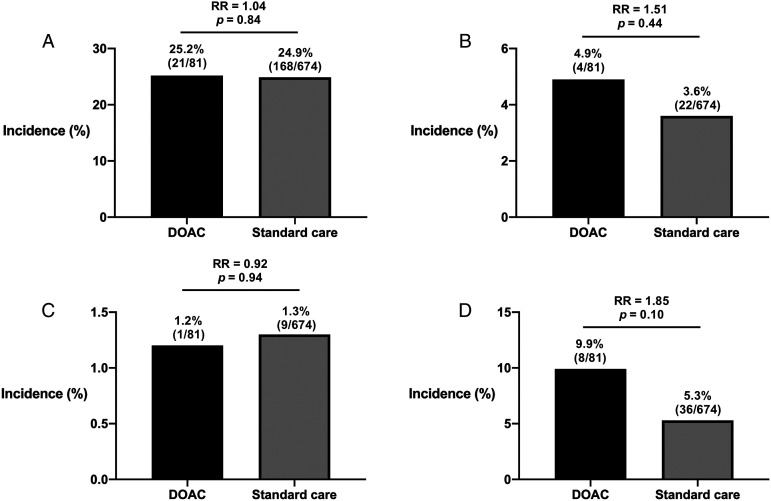
Outcome measures (a) blood transfusion (b) haemorrhage (c) venous thromboembolism (d) all-cause mortality.

Pre-operative haemoglobin levels were comparable between the DOAC and standard treatment groups (Table 2). Change in haemoglobin levels pre- and post-operatively were similar, no differences in post-operative haemoglobin levels on the first day and nadir within the first week. Subgroup analysis of patients undergoing surgery within 24 hours of the last DOAC dose showed a greater drop in pre- and post-operative nadir haemoglobin levels compared to the rest of the study population, although this was not statistically significant. The incidence of blood transfusion was 25.2% in the DOAC group and 24.9% in the standard treatment group (RR: 1.04, 95% CI: .70-1.54, P = .84) (Figure 1). Haemorrhage occurred in 4.9% of patients in the DOAC group and 3.6% of patients in the standard treatment group (RR: 1.51, 95% CI: .53–4.28, P = .44) (Figure 1). The incidence of VTE in the DOAC and standard treatment groups were 1.2% and 1.3%, respectively (RR: .92, 95% CI: .12–7.20, P = .94) (Figure 1). All-cause mortality after 30 days was 9.9% in the DOAC group and 5.3% in the standard treatment group (RR: 1.85, 95% CI: .89–3.84, P = .10) (Figure 1). Subgroup analysis did not reveal any differences in risk of blood transfusion, haemorrhage, VTE and mortality in patients undergoing surgery within 24 hours of the last DOAC dose with the rest of the cohort. Multivariate logistic regression modelling found no effect of the timing of surgery on any of the primary outcome measures.

**Table 2.** Outcome measures.

Outcome	DOAC Group (n = 81)	DOAC Group, <24 hours (n = 19)	DOAC Group, ≥24 hours (n = 62)	Standard Treatment Group (n = 674)	*P-*value
Pre-operative haemoglobin – g/L	123.1 ± 17.8	119.3 ± 16.0	124.2 ± 18.3	121.0 ± 16.5	.33
Post-operative haemoglobin – g/L Day 1 Week 1 nadir	103.3 ± 17.8 95.4 ± 17.5	100.3 ± 14.5 87.8 ± 14.8	104.3 ± 18.7 97.8 ± 17.7	101.6 (17.6) 94.9 (17.4)	.430.80
Change in haemoglobin – g/l pre-op – Day 1 Pre-op – Week 1 nadir	19.5 ± 13.5 27.3 ± 16.1	19.0 ± 16.5 31.5 ± 20.2	19.6 ± 12.5 26.0 ± 14.5	19.6 ± 14.0 26.9 ± 16.6	.950.84
Blood transfusion (%)	21 (25.2)	6 (31.6)	15 (24.2)	168 (24.9)	.84
Haemorrhage (%)	4 (4.9)	1 (5.3)	3 (4.8)	22 (3.6)	.43
Venous thromboembolism (%) Pulmonary embolism Deep vein thrombosis	1 (1.2) 1 (1.2) 0 (.0)	0 (.0) 0 (.0) 0 (.0)	1 (1.2) 1 (1.2) 0 (.0)	9 (1.3) 9 (1.3) 0 (.0)	.94
All-cause mortality (%)	8 (9.9)	2 (10.5)	6 (9.7)	36 (5.3)	.10

## Discussion

In the present study, changes in pre- and post-operative haemoglobin levels, incidence of blood transfusion and rates of peri- and post-operative haemorrhage were equivocal in patients receiving DOAC treatment compared to patients who were on standard care with LMWH. These findings are consistent with earlier studies that have reported comparable peri-operative blood loss in patients on DOAC therapy compared to patients who were not on any anti-coagulation, albeit with greater delays in surgery.^14–^^16^ In contrast, DOAC use was not associated with delays in surgery in this study. Furthermore, analysis of our patient cohort revealed that the timing of surgery after the last DOAC dose had no impact on bleeding outcomes, which builds on previous work by Mullins et al.^17^ The present study advances this further as by analysing data based on the timing of surgery from the last DOAC dose instead of admission.

There was a greater drop in pre- and post-operative nadir haemoglobin levels within the first week in DOAC group who underwent surgery within 24 hours of their last dose, although this was non-significant. Nagra et al have previously suggested that the nadir haemoglobin occurs 5 days post-operatively following hip fracture surgery.^18^ This is supported by our data, which estimates the nadir haemoglobin level to occur between three to 5 days post-operatively, with no difference between patients receiving DOACs or standard care. As such, previous studies that have utilised post-operative haemoglobin levels on the first post-operative day may have underreported the estimated amount of blood loss.^15–^^17^ Whether this has any meaningful clinical impact is unclear. Nevertheless, our findings warrant further evaluation in a larger cohort to elucidate the safety of early surgery in these patients.

The risk of VTE was similar between patients on DOAC therapy and patients with no anti-coagulation. Recent studies have suggested that DOACs are as effective as LMWH in preventing VTE following surgery for hip fracture, without an increasing risk of haemorrhage.^19–^^21^ In patients on existing treatment, the duration of interruption is unclear. The current consensus is that the DOAC should be resumed after at least 24 hours post-operatively, once haemostasis has been achieved.^22,^^23^ All-cause mortality in the DOAC and standard treatment groups were 9.9% and 5.3%, respectively. The higher mortality rate in the DOAC group can be attributed to the greater pre-operative co-morbid status of these patients. Nonetheless, our data is consistent with cohort and registry studies, which suggests that the study population is comparable.^5,^^24^ None of the deaths in our study were attributed to haemorrhage or VTE.

Several limitations must be considered in the present study. Due to the retrospective design, there are inherent biases and confounding factors that could potentially influence our results. However, sensitivity analyses performed did not identify any meaningful effect size arising from differences between the two cohorts. Pre-operative blood DOAC levels were not obtained so no correlations could be made with the outcomes measured. Nevertheless, this is reflective of the real world setting where measurements of blood DOAC levels are not readily available. The a priori estimate of sample size increases the likelihood of detecting of any meaningful clinical differences between the two groups. However, it is possible that the subgroup analysis of the cohort of patients undergoing surgery within 24 hours of their last DOAC dose is susceptible to a Type II error due to the small size in this group.

## Conclusion

This study builds on growing evidence that surgery for hip fracture in patients on DOAC therapy is not associated with an excessive risk of haemorrhage, irrespective of the timing of surgery. Timely surgical fixation of the hip fracture in this population is indicated in the absence of other risk factors for haemorrhage. We acknowledge that there will be a population of patients who have a higher predisposition towards haemorrhagic events, which will require further evaluation in future work.

## Supplemental Material

sj-pdf-1-gos-10.1177_21514593221088405 – Supplemental Material for Timing of Surgery for Hip Fracture in Patients on Direct Oral Anti-coagulants: A Population-Based Cohort Study Click here for additional data file. Supplemental Material, sj-pdf-1-gos-10.1177_21514593221088405 for Timing of Surgery for Hip Fracture in Patients on Direct Oral Anti-coagulants: A Population-Based Cohort Study by En Lin Goh, BSc (Hons), MBBS (Dist), MRCS, Swathikan Chidambaram, Suprabha Rai, Angela Kannan and Sambandam Anand in Geriatric Orthopaedic Surgery & Rehabilitation
